# Comparison of three-dimensional printing guides and double-layer guide plates in accurate bracket placement

**DOI:** 10.1186/s12903-020-01110-w

**Published:** 2020-04-28

**Authors:** Yue Zhang, Chunhao Yang, Yanfeng Li, Dong Xia, Tingting Shi, Changjian Li

**Affiliations:** Department of Stomatology, the Fourth Medical Center, Chinese PLA General Hospital, No.51 Fucheng Road, Haidian District, Beijing, 100048 China

**Keywords:** Indirect bonding, Double-layer guide plate, Three-dimensional printing guide, Accuracy, Bracket placement

## Abstract

**Background:**

In the current study, we aimed to evaluate the accuracy of indirect bonding by either *three-dimensional (3D) printing guides* or *double-layer guide plates.* The results may serve as a clinical reference for bracket placements.

**Methods:**

In total, 140 teeth were collected and arranged into five pairs of full dentition. The marking points were labeled on the buccal/labial surface of the crown in these orthodontic study models. (1) *3D printing guide*: A digital profile was generated using an intraoral scanner. Two types of indirect bonding guide, namely the whole denture type and the single tooth type, were designed with the 3Shape TRIOS® Standard intraoral scanner and fabricated using 3D printing technology. (2) *Double-layer guide plate*: A working model was obtained by replicating the experimental models, and the *double-layer guide plate* was then made of the inner layer soft film (1.0 mm thickness) and the outer layer hard film (0.6 mm or 0.8 mm thickness). Brackets were transferred from working models to study models by the indirect bonding trays. We measured and analyzed the distance between marking points and bracket placement. Statistical analysis was done using SPSS 20.0 software. The accuracy of indirect bonding between *3D printing guide* and *double-layer guide plate* was compared using paired *t*-test.

**Results:**

According to our data, there was a significant difference between the 0.6 mm group and 0.8 mm group when the brackets were indirectly adhered using *double-layer guide plates* (*p =* 0.036). However, no statistical significance in bracket positioning accuracy was revealed between two types of *3D printing guide* (*p* = 0.078), as well as between the *3D printing guide* group and the 0.6 mm *double-layer guide plate* group (*p* = 0.069).

**Conclusions:**

When applying *double-layer guide plates* for indirect bonding, the 0.6 mm group is more accurate than the 0.8 mm group. When utilizing *3D printing guides* for indirect bonding, whole denture type is more accessible than single tooth type but with no significant difference in accuracy. The accuracy of indirect bonding is comparable when using *3D printing guides* (whole denture type) and *double-layer guide plates* (0.6 mm).

## Background

The positioning and bonding of orthodontic brackets significantly affect the clinical outcome and is the most critical step in orthodontic treatment [1]. Orthodontic brackets can be adhered to the tooth surfaces by direct or indirect bonding. Due to the presence of saliva and some inaccessible tooth positions, direct bonding usually takes longer chair-side time and lacks accuracy [2]. To optimize the accuracy of bracket positioning, indirect bonding technique was proposed by Silverman and Cohen in 1972 and since then became a popular alternative [1, 3]. It has been shown that errors associated with bracket positioning were minimized in the aspects of height, mesiodistal position, and angulation when using indirect bonding [4]. There are two stages in the indirect bonding procedure, the laboratory stage and the clinical stage [2]. After obtaining patients’ orthodontic models, brackets are bonded to the study models in the laboratory stage, and then placed on tooth surfaces integrally with the aid of a customized transfer tray in the clinical stage [5, 6]. Though the chair-side time is significantly reduced, there are considerable laboratory-associated expenses when indirect bonding is chosen [7].

With the increasing applications of indirect bonding, various designs of transfer trays and novel technologies are implemented in the treatment procedure. In the laboratory stage, the patients’ occlusal interrelationship can be duplicated either by impression or digital scanning. The former is a traditional method to generate double-layer guide plates; though with a lower cost, this method typically takes longer laboratory time and is susceptible to human errors. The latter is incorporated with cutting-edge 3D printing technology that provides various advantages, such as precise 3D images, convenience in file storage, and accuracy in image analysis and outcome prediction [5]. Currently, there is limited information on the comparison between traditional indirect bonding and digital indirect bonding in bracket positioning accuracy. To investigate differences in bracket positioning accuracy, we designed different types of transfer trays by traditional impression or 3D printing technology, and then performed a comprehensive evaluation of the clinical efficacy of each design. The data will provide valuable clinical guidance for bracket placements.

## Methods

### Fabrication of study models

**Fig. 1:**
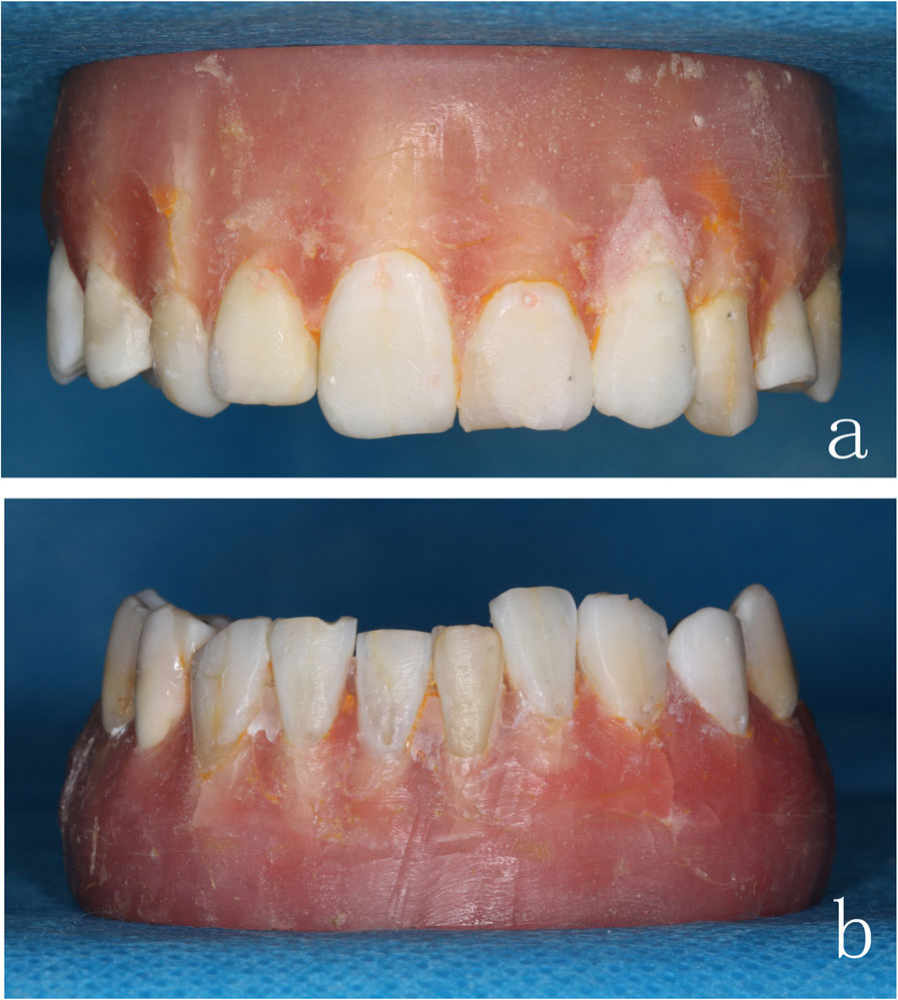
The study model. **a** Maxillary model with marking points. **b** Mandibular model with marking points

We collected 140 teeth with normal crown morphology and no evident defects or restorations at the buccal/labial surfaces, and then sterilized them for the following study. The teeth were arranged into five pairs of complete dentition with mild malocclusion but no torsion/tilting/overlapping. The roots were embedded in denture-base self-curing resin with crowns exposed. The marking points were labeled at the mid of the cervical third and distal end of the incisal third on the study models (Fig. 1).

### Production of *3D printing guides* and indirect bonding procedure

**Fig. 2:**
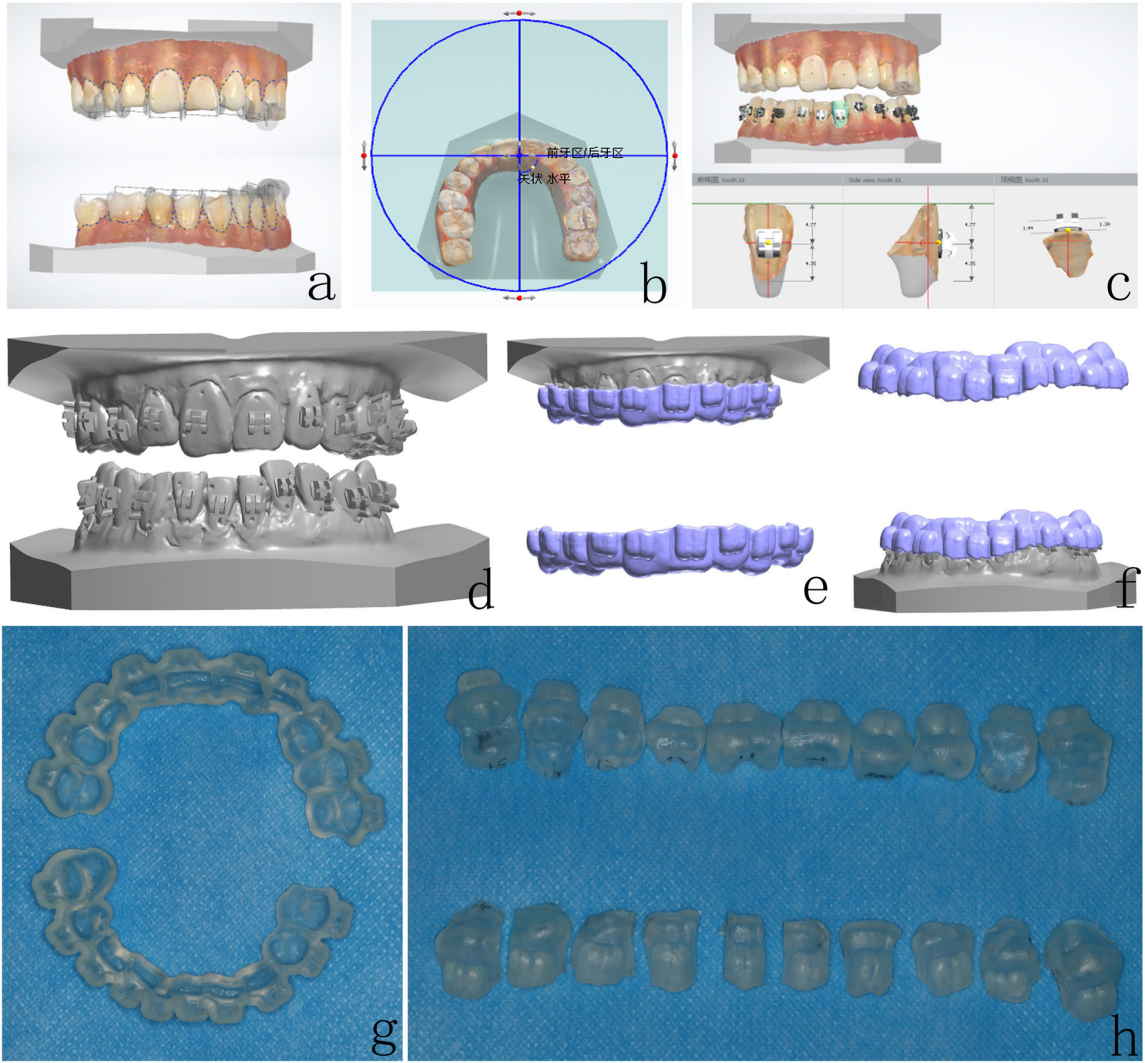
Digital design and *3D printing guides*. **a** Distinguishing teeth and gingiva on the digital models. **b** Establishing the occlusal plane. **c** Adjusting the positioning of brackets. **d** Simulation of bracket positioning. **e**-**f** Guide plate for indirect bonding on the digital models. **g***3D printing guide* – whole denture type. (h) *3D printing guide* – single tooth type

Digital models were generated using 3Shape TRIOS® Standard intraoral scanner (Fig. 2a). 3Shape OrthoAnanlyzer™ software was applied to label the marginal gingiva and establish the axis and center of individual crowns, as well as the occlusal plane (Fig. 2b-c). The positioning of the brackets was determined on the digital 3D model (Fig. 2c-f). Finally, guide plates were generated by 3D printing technology (Project 3510 DP) as the whole denture type (Fig. 2g) and single tooth type (Fig. 2h).

**Fig. 3:**
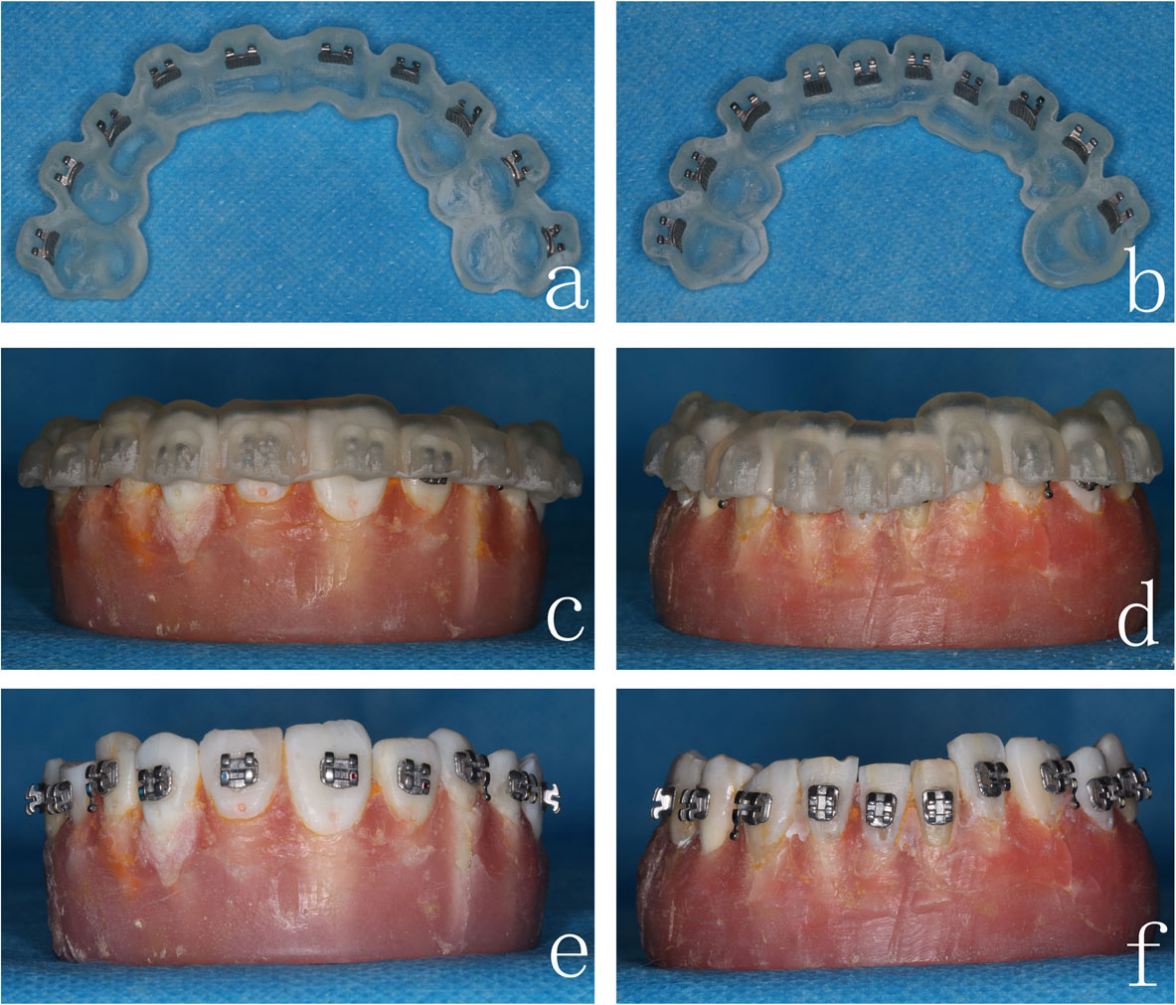
*3D printing guide*s and indirect bonding procedure. *3D printing guide* of the **a** maxillary and **b** mandibular dentitions. *3D printing guides* are placed on the **c** maxillary and **d** mandibular study models. Completion of bracket positioning on the **e** maxillary and **f** mandibular study models

Before the indirect bonding procedure, we polished the tooth surfaces, etched buccal/labial surfaces of the crowns for 15 s, and isolated the area with cotton rolls and dried xx thoroughly. The brackets were positioned in the *3D printing guides* (the whole denture type or the single tooth type), and 3 M Unitek Transbond™ XT light-curable adhesives were applied to the base of the brackets (Fig. 3a-b). The *3D printing guides* were then placed on the study models, and each border of the brackets was light-cured for 5 s (Fig. 3c-d). Thereafter, the *3D printing guides* were removed to clean excess adhesives around the brackets (Fig. 3e-f).

### Production of *double-layer guide plates* and indirect bonding procedure

**Fig. 4:**
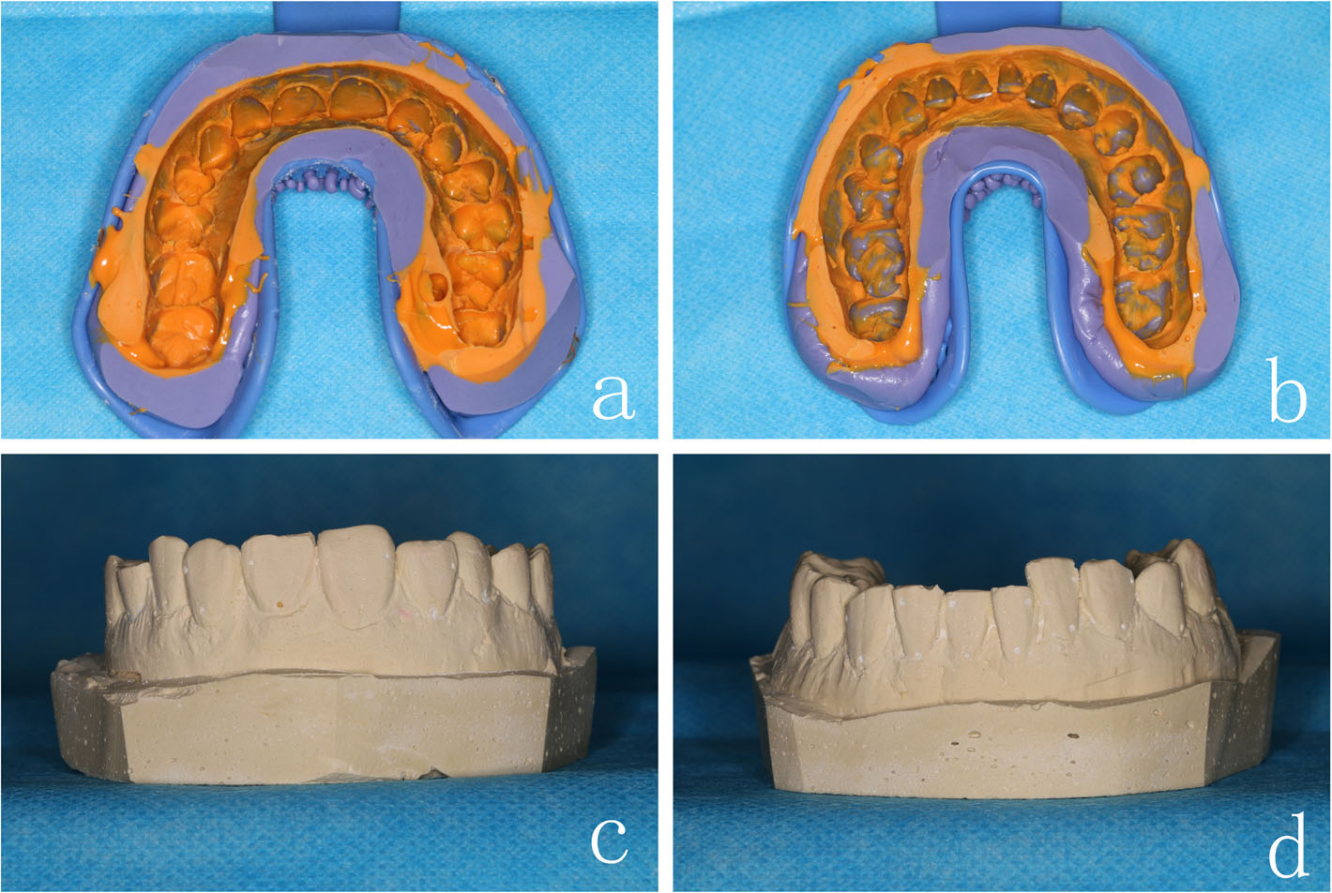
The impression of **a** maxillary and **b** mandibular dentitions, and the plaster casts of **c** maxillary and **d** mandibular dentitions

**Fig. 5:**
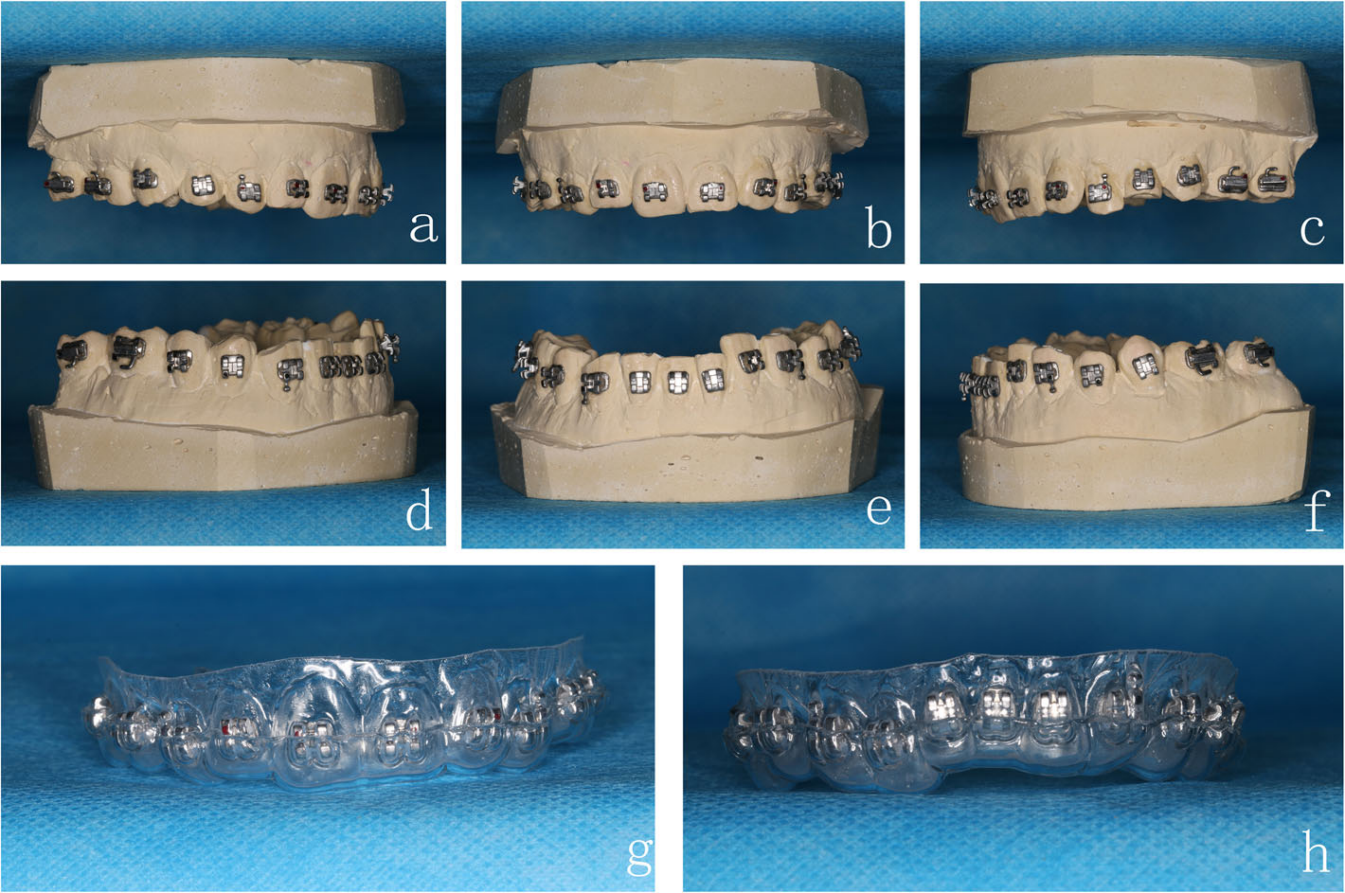
Bracket positioning on the plaster casts and production of *double-layer guide plates.* Brackes positioning on the **a**-**c** maxillary and **d**-**f** mandibular dentitions. *Double-layer guide plate* of the **g** maxillary and **h** mandibular dentitions

A precise impression of the working models with marking points was obtained using silicone-based impression materials (Fig. 4a-b). After 24-h crystallization at the room temperature, plaster casts were duplicated from the silicone molds (Fig. 4c-d). A thin layer of separation agents was applied to the cast tooth surfaces; then, the brackets were positioned and adhered on the crowns using 3 M Transbond™ XT light-curable adhesives and light-cured for 5 min (Fig. 5a-f). *Double-layer guide plates* were manufactured by Erkoform-3D Thermoformer with a 1 mm inner layer (soft film) and 0.6 mm or 0.8 mm outer layer (hard film). Lastly, we trimmed the excess materials of the inner layer to 2 mm above the crowns and the outer layer until covering 2/3 of the brackets (Fig. 5g-h).

**Fig. 6:**
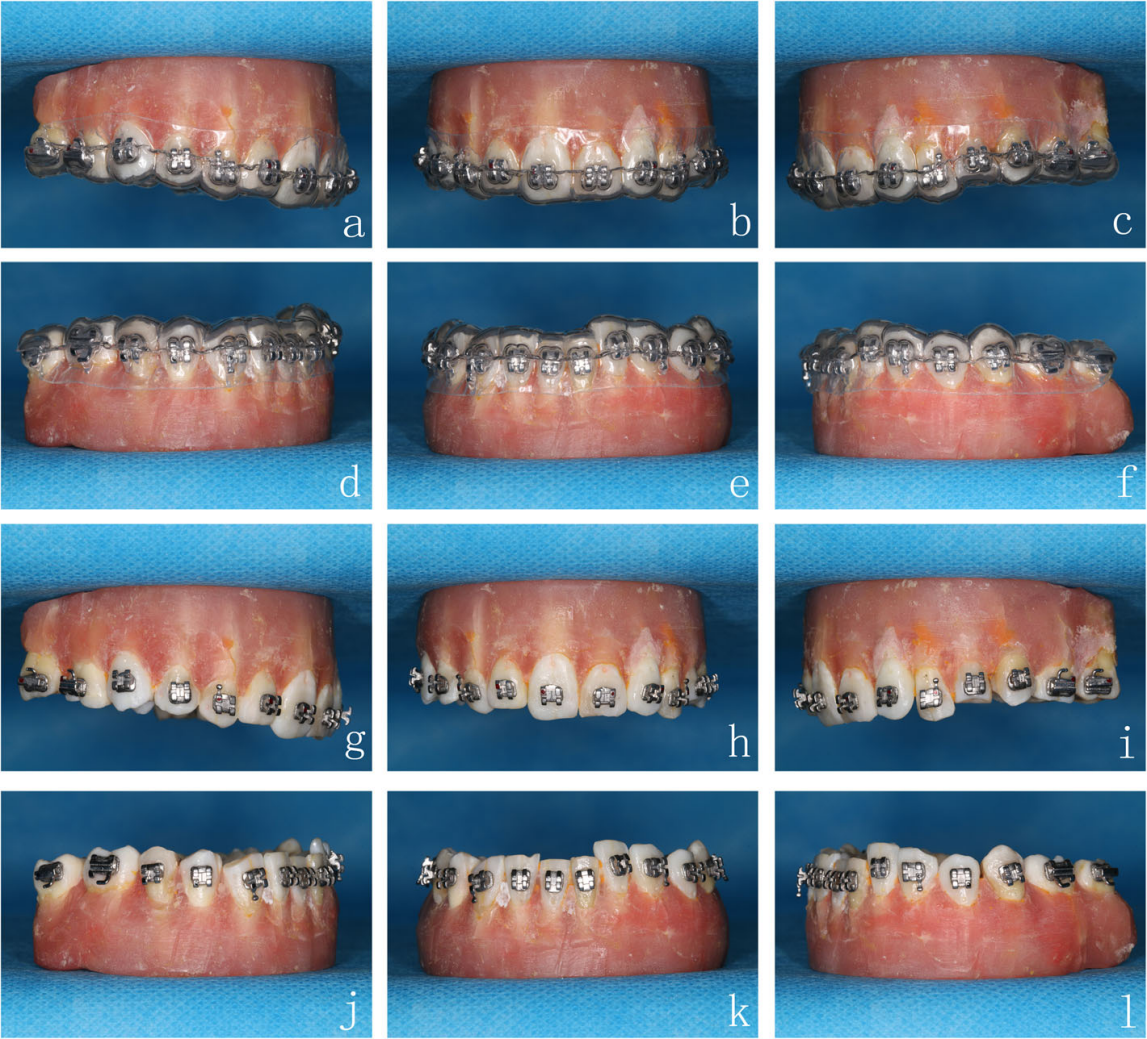
*Double-layer guide plates* and indirect bonding procedure. *Double-layer guide plates* are placed on the **a**-**c** maxillary and **d**-**f** mandibular study models. Completion of bracket positioning on the **g**-**i** maxillary and **j**-**l** mandibular study models

Before the indirect bonding procedure, we polished the tooth surfaces, etched buccal/labial surfaces of the crowns for 15 s, and isolated the area with cotton rolls and dried xx thoroughly. 3 M Sondhi™ Rapid-Set Indirect Bonding Adhesive was used for the indirect bonding procedure. The brackets were positioned in the *double-layer guide plates* (0.6 mm or 0.8 mm outer layer). Solution A was applied to the tooth surfaces, and solution B was applied to the base of brackets. The *double-layer guide plates* were then placed on the study models (Fig. 6a-f). After fixation for 2 min, the outer hard layer was taken off, followed by the inner soft layer. The excess adhesives around the brackets were then removed carefully (Fig. 6g-l).

### Measurements

**Fig. 7:**
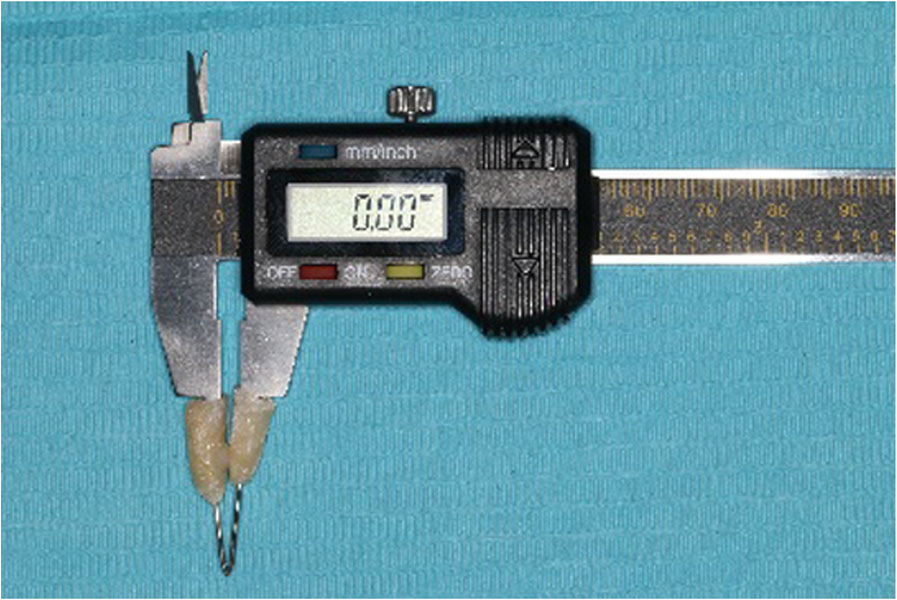
Electronic caliper

**Fig. 8:**
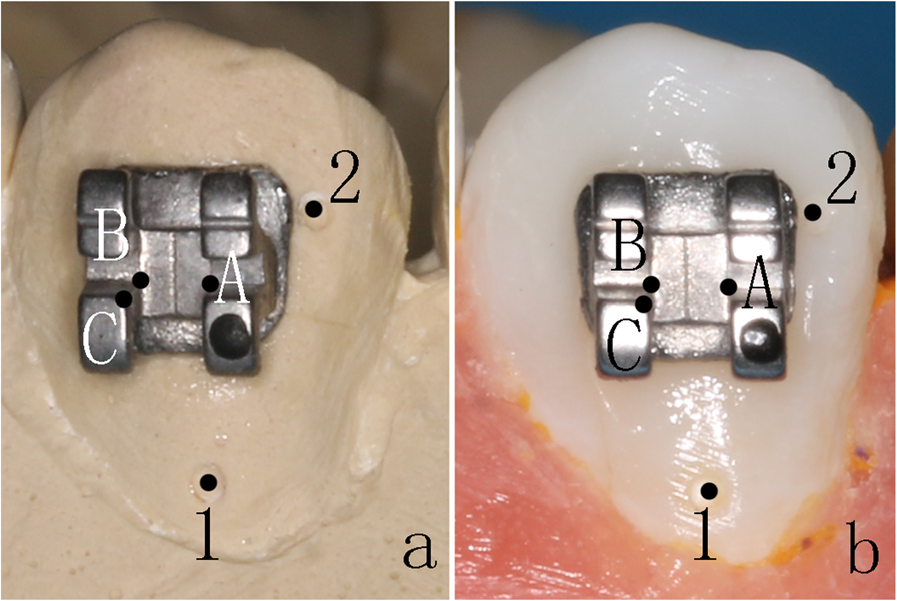
The marking points on the plaster cast and study model

In the *3D printing guide* group, Mimics software was applied to measure the distance between the bracket positions and marking points on the crowns in the digital models. Three measurements were completed and recorded for both designs (i.e., whole denture type and single tooth type). In the *double-layer guide plate* group, electronic calipers (Fig. 7) were used to measure the distance (d_A1_, d_B1_, d_B2_, and d_C2_) between the bracket positions and marking points on the crowns in the study models (Fig. 8). Three measurements were completed and recorded for both designs (i.e., 0.6 mm outer layer and 0.8 mm outer layer).

### Statistical analysis

Statistical analyses were conducted using *SPSS* 20.0 software. The accuracy of indirect bonding between *3D printing guide* and *double-layer guide plate* was compared using the paired *t*-test. *P* < 0.05 indicated statistical significance.

## Results

As shown in Table 1, in the *3D printing guide* group, there was no statistical significance in bracket positioning accuracy between the whole denture type and single tooth type (*p* = 0.078). In the *double-layer guide plate* group, when bracket positioning accuracy was compared using different thicknesses of the outer layer (0.6 mm vs. 0.8 mm), the 0.6 mm type demonstrated better results (*p* = 0.036), as shown in Table 2.

**Table 1:** The comparison of different designs in the *3D printing guide* group

	Single tooth type	Whole denture type	*p*
Distance (*x ± s*, mm)	0.22 ± 0.05	0.21 ± 0.05	0.078

*p* < 0.05

**Table 2:** The comparison of different designs in the *double-layer guide plate* group

	0.6 mm outer layer	0.8 mm outer layer	*p*
Distance (*x ± s*, mm)	0.22 ± 0.08^*^	0.24 ± 0.08	0.036

^*^p < 0.05

We then further compared the accuracy of indirect bonding between *3D printing guides* (whole denture type) and *double-layer guide plates* (0.6 mm), the results were comparable between two groups (*P* = 0.069) (Table 3). However, indirect bonding using *double-layer guide plates* (0.6 mm) cost less chair-side time than the *3D printing guides* group.

**Table 3:** The comparison of bracket positioning accuracy between *3D printing guide* and *double-layer guide plate*

	*Double-layer guide plate* (0.6 mm)	*3D printing guide* (Whole denture type)	*p*
Distance (*x ± s*, mm)	0.22 ± 0.08	0.21 ± 0.05	0.069

*p* < 0.05

## Discussion

Accurate positioning and bonding of brackets impact on the outcome of orthodontic treatments [1]. In comparison with direct placement of brackets, the indirect bonding procedure significantly decreases chair-side time and improves patient comfort [8]. Indirect bonding was reproducibly demonstrated to be more accurate than direct bonding on bracket positioning with less torque error and rotation deviation [9, 10]. Furthermore, indirect bonding reduces plaque accumulation and decalcifies white spots around the orthodontic brackets [11]. Though indirect bonding technique becomes popular in orthodontic treatment, there are still some disadvantages, such as time-consuming and technique-sensitive laboratory procedures and additional expenses on materials [7]. There are many types of transfer trays for indirect bonding, such as *double-layer guide plates* and *3D printing guides*. Overall, *double-layer guide plates* exhibit shorter time for both fabrication and clinical bonding.

The concept of *double-layer guide plates* for indirect bonding was initially proposed in 1990s; thermoplastic and silicone-based materials are commonly used to produce transparent transfer trays [12, 13]. *Double-layer guide plates* are made on super-hard plaster casts with the aid of thermoformers [7]. Both silicone-based impression material and super-hard plaster exhibit optimal stability, with a deformation rate of 0.05% in the former and 0.1% in the latter [7]. Typically, the outer layer is rigid to ensure the stability of bracket positioning, and the inner layer is soft to ease removal after bracket transfer. In our preliminary studies, we tested different thicknesses of outer and inner layers. The results revealed that 1 mm soft film as the inner layer with either 0.6 mm or 0.8 mm hard film as the outer layer allowed the best accuracy and stability. Therefore, in the following experiments, we adopted the setting to manufacture *double-layer guide plates*.

3D printing is an additive manufacturing (AM) and rapid prototyping (RP) technology [14]. The first 3D printing machine was introduced in 1986 and incorporated with stereolithography appearance (SLA), selective laser sintering (SLS), fused deposition modeling (FDM), and laminated object manufacturing (LOM) [14]. The 3D printing technology is highly flexible and customizable, which allows timely production of individualized subjects [14]. Meanwhile, it has a high resolution for detailed designs (~ 0.01 mm horizontally and ~ 0.2 mm vertically) [14]. With all these features, 3D printing is now widely applied in dentistry, in particular, to generate customized brackets, orthodontics models, and guide plates [14]. In the current study, we used 3D printing technology to design two indirect bonding guide plates, whole denture type and single tooth type.

Multiple methodologies have been applied to evaluate the transfer accuracy of indirect bonding. Digital photography or CBCT was performed to capture the images of study casts and compare bracket positioning before and after the transfer [10, 15, 16]. Based on the study design by Castilla et al., the linear distance (mesiodistal, buccolingual, vertical) and angular differences between the intended and actual bracket position were measured and recorded for further analyses [16, 17]. In the current study, we aimed to investigate bracket placement accuracy with different indirect bonding guide plates, namely *double-layer guide plates* and *3D printing guides*. According to our preliminary studies for the *double-layer guide plates* and *3D printing guides*, the positioning discrepancy after the brackets transfer was − 0.022 mm ± 0.089 mm and − 0.025 mm ± 0.077 mm, respectively. With the setting of α = 0.05, Z_0.05/2_ = 1.96, and β = 0.20, Z_0.20_ is 0.842. To ensure 95% confidence interval and power of 0.8, we will need at least 128 (*double-layer guide plates*) and 75 teeth (*3D printing guides*) for comparison ($\mathrm{N}={\left[\frac{\left({Z}_{\alpha }+{Z}_{\beta}\right){\sigma}_d}{\delta}\right]}^2$). To eliminate potential confounding factors, we collected 140 teeth and arranged them into five pairs of full dentition that exhibited mild malocclusion. We measured the distance between brackets and marking points on the tooth surfaces before and after bracket transfer. Paired *t*-test was performed to compare transfer accuracy between *3D printing guides* and *double-layer guide plates.*

Our study demonstrated a significant difference between the 0.6 mm group and 0.8 mm group when using *double-layer guide plates* (*P* < 0.05), with the 0.6 mm group exhibiting a better transfer accuracy. *Double-layer guide plates* were produced by a thermoformer, and the thickness of films is proportional to the processing time. When *double-layer guide plates* are manufactured, both the impression material and plaster have some elasticity, which may lead to subtle transformation and clinical inaccuracy. Therefore, the long processing time negatively affects the transfer accuracy of guide plates. On the other hand, the design of *3D printing guides* was completed digitally and printed directly with no material deformation involved in the entire process. However, the accuracy of *3D printing guides* is greatly affected by the collection of digital images and file transformation. With minimized human errors, both whole denture type and single tooth type of *3D printing guides* exhibited optimal bracket transfer accuracy. The single tooth type needs to be bonded individually, while the whole denture type can be delivered more efficiently. With the aid of computer software, *3D printing guides* has numerous advantages. For example, CBCT images of roots and jawbone morphology can be incorporated into the treatment plan to avoid adverse events of orthodontic treatments (e.g.*,* unparalleled roots and dehiscence). This will enhance the predictability and accuracy of the clinical outcome.

Interestingly, the transfer accuracy of indirect bonding is comparable when using *3D printing guides* (whole denture type) and *double-layer guide plates* (0.6 mm). Though no statistical significance was shown in our data, the overall discrepancy before and after bracket transfer was lower in the *3D printing guides* group*.* This finding might be due to our in vitro study models with only mild malocclusion. Further in vivo studies in more severe clinical cases, such as malocclusion with torsion/tilting/overlapping, will be essential to investigate the efficacy and generalizability of *3D printing guides* and *double-layer guide plates*.

## Conclusion

The accurate positioning and bonding of brackets set the foundation for effective orthodontic treatments [1]. The indirect bonding technique using either *double-layer guide plate* or *3D printing guide* sufficiently increases the accuracy of bracket placement. *Double-layer guide plate* is a conventional strategy with shorter fabrication time. On the other hand, *3D printing guide* combined with digital imaging software demonstrates many advantages and is gaining more and more popularity in orthodontic treatments. In the current study, we utilized five pairs of dentition with mild malocclusion and demonstrated that both guide plates have similar accuracy for bracket transfer. In particular, double-layer guide plates with the 0.6 mm outer layer exhibited better bracket transfer accuracy than the 0.8 mm group. The study was done in vitro without considering the impact of the tongue, the buccal/labial mucosa, and saliva. Therefore, it is essential to have a well-designed in vivo study to address other physiological factors.

## Data Availability

The datasets generated and analyzed during the current study are available from the corresponding author on reasonable request.

## Abbreviations

3D: Three-dimensional
SLA: Stereolithography apparatus
SLS: Selective laser sintering
FDM: Fused deposition modeling
LOM: Laminated object manufacturing
AM: Additive manufacturing
RP: Rapid prototyping
CBCT: Cone beam computed tomography
